# Improving Clinical Nurses' Development of Supervision Skills through an Action Learning Approach

**DOI:** 10.1155/2020/9483549

**Published:** 2020-02-19

**Authors:** Irene Sommer, Karin Larsen, Carsten M. Nielsen, Britta V. Stenholt, Ida Torunn Bjørk

**Affiliations:** ^1^Department of Cardiology, Aarhus University Hospital, 8200 Aarhus N, Aarhus, Denmark; ^2^Department of Endocrinology, Aarhus University Hospital, 8200 Aarhus N, Aarhus, Denmark; ^3^VIA University College, Nurse Education, 8200 Aarhus N, Aarhus, Denmark; ^4^VIA University College, Nurse Education, 8600 Silkeborg, Denmark; ^5^Department of Nursing Science, Institute of Health and Society, University of Oslo, 0318 Oslo, Norway

## Abstract

The aim of this study was to investigate action learning as an implementation method in a large-scale project with many participants in several autonomous and geographically spread groups. The focus of the implementation was the Model of Practical Skill Performance as a learning and supervision tool in the clinical education of nursing students. Nineteen action learning groups were established, and a total of 129 clinical supervisors and 13 facilitators were involved. To evaluate the implementation process, qualitative data were generated through three focus group interviews, questionnaires, and notes. Data illuminate clinical supervisors' perceptions of value, impact, and sustainability when they participate in an action learning group to become familiar with the Model of Practical Skill Performance. The deductive data analysis was guided by central concepts from action learning. Action learning proved to be an engaging and effective tool in the implementation where the main strength seemed to be the autonomous local group supporting collective reflections on actions. Clinical supervisors had the right competences to adopt a reflective process-oriented approach, which is the hallmark of action learning. This study shows the necessity of collaboration between stakeholders in practice, education, and management to implement large-scale projects in clinical practice. The findings imply that managers should choose participants on the basis of their motivation and their voluntary wish to participate and that nurses' immersion in the project over time aids implementation.

## 1. Introduction

It is a challenge to ensure that newly qualified nurses have sufficient competences in performing practical nursing skills [1–3]. A plethora of practical skills in clinical practice require both instruction and training, but only some of these are introduced and practiced during the nursing education program.

Clinical placements in nursing education provide students with varied but also limited opportunities to practice, train, and perform practical skills in a health sector characterized by a high degree of specialization and introduction of new medical technologies [4]. Patients present with medical illnesses and symptoms that are difficult to imitate in a simulated setting, and they represent interesting, complex learning situations for nursing students [5, 6]. Clinical supervisors thus play an important role in securing students' learning, helping them to build on previously acquired skills and knowledge, to obtain new skills, and to introduce models on how to become a proficient nurse [7]. At the same time, the supervisors have to ensure that learning activities are safe for both students and patients [8, 9]. Several studies highlight that nurses lack supervision skills [9–13] and that organization of the supervision could be improved [9].

To meet the challenges of supervision during the acquisition of practical skills in clinical practice, the group Research in Nursing Skills (RiNS; http://www.rins.dk) developed a supervision tool (Table 1) based on the normative, theoretical, and empirical Model of Practical Skill Performance [14]. The model was originally presented in a doctoral dissertation, aiming to show the complexity of practical skill performance in nursing [15, 16]. It was based on educational and caring theory, existing research on practical skill learning in nursing education, and empirical data from video recordings of nurses performing practical skills in the clinical setting [15, 16]. The supervision tool further developed by RiNS includes definitions of six central components of any practical nursing skill: substance, sequence, accuracy, fluency, integration, and caring comportment, as well as quality criteria for the evaluation of the performance of these elements [17]. The relevance and benefit of the supervision tool in formative assessment, supervision, and reflection have been tested in nursing homes, hospital wards, and simulated settings by nursing students, clinical supervisors, and faculty [18–21]. Since 2013, the model has been included in the nursing curriculum for 1500 annual students at VIA University College in Denmark, where the present study was conducted.

The Model of Practical Skill Performance has been developed and tested as a generic tool for learning, supervision, and formative evaluation of practical skill performance and has successfully been implemented in simulated settings and in small-scale clinical practice projects [18–21]. However, we experienced that widespread implementation of the tool was a challenge during students' clinical placement. Thus, a structured action learning approach was launched in 2013-2014 as a project that aimed to support a large number of clinical supervisors to implement the supervision tool in clinical practice.

Clinical supervisors are nurses working in clinical departments, and although many of them are familiar with the model, its use in clinical supervision is limited. Previous small-scale implementation studies using an action learning approach have been shown to be effective in strengthening implementation in local settings with few participants [18, 19]. Action learning has promoted implementation processes in several studies within the health care field, such as improvement of skills in clinical decision-making and leadership [22–24].

The present study investigated the usefulness of a structured action learning approach as a method to implement the Model of Practical Skill Performance [15, 16], a tool to support students during practical skill learning in nursing [18].

### 1.1. Aim and Research Question

The aim of this study was to investigate the impact of action learning as an implementation method in a large-scale project with many participants in several autonomous and geographically spread groups. The following research question was formulated: What are clinical supervisors' perceptions of value, impact, and sustainability when they participate in an action learning group to become familiar with the Model of Practical Skill Performance and its use as a learning and supervision tool?

## 2. Methods

This study has an exploratory and mainly qualitative research design [25] to illuminate participants' perceptions of attending an action learning project.

### 2.1. Setting and Samples

This study was conducted in Central Denmark Region, one of the five administrative units in Denmark responsible for the running of hospitals. The project leaders were faculty or clinical educators at VIA University College that offered the bachelor program in nursing for this region and were members of the research group RiNS. This study covered six hospitals, 19 municipalities, and six nursing education campuses.

The participants were clinical supervisors and facilitators included in the project through an open e-mail invitation in autumn 2013 and spring 2014. Participation was generally voluntary; however, a few nurses participated upon direct request from their manager. A total of 129 clinical supervisors and 13 facilitators were included. All participants were female. The clinical supervisors were experienced nurses with at least six weeks of pedagogical education (equivalent to 10 ECTS points), and several had previously participated in group-based nursing supervision. Their knowledge about the Model of Practical Skill Performance varied from little knowledge to some degree of certainty in applying the model as a supervision tool. Most of the facilitators had a master's degree and had skills in facilitating individual- and group-based reflective learning processes. Most of the facilitators were clinical nurses responsible for the clinical education environment in hospitals or municipalities.

### 2.2. Intervention

**Figure 1:**
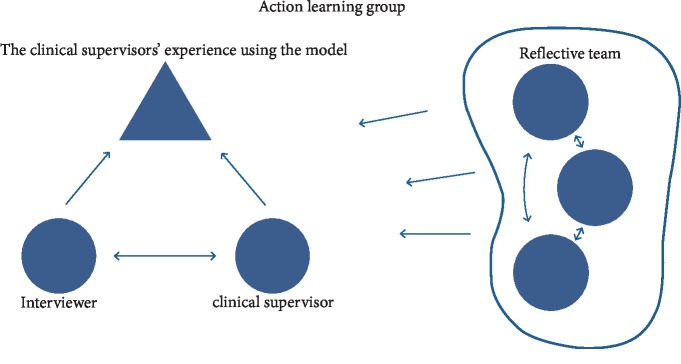
Structured reflection process in the action learning group.

The primary intervention used in the implementation process of the Model of Practical Skill Performance was action learning. The idea in action learning is that the participants' skills develop in mandatory reflective action learning groups while participants obtain understanding by doing and develop when they are capable of doing on the basis of a better understanding [26]. Madsen and Birkelund [27] described a framework of action learning design inspired by systemic therapy and nursing supervision, including a case presenter, an interviewer, and a reflective team. All facilitators participated in a seminar where they practiced skills by leading reflection in action learning groups and acting as interviewers, as illustrated in Figure 1. The key elements were contextual learning gained from practice, collective reflection, feedback, encouragement, and a psychologically safe environment created for learning [22, 27, 28].

At a kick-off seminar, all clinical supervisors and facilitators were introduced to the action learning framework including the structure of the action learning groups (Figure 1). Furthermore, the Model of Practical Skill Performance was presented by the creator of the model, and experiences from using the model in clinical supervision were presented.

Facilitators were responsible for establishing action learning groups, organizing meetings, maintaining the action learning framework in the group, and keeping contact with the project leaders. Nineteen action learning groups were formed. Six of the facilitators worked with two groups each. Each group included 5–10 members from one geographical area. The action learning groups held 4-5 meetings during the six-month course, and meetings were structured with agreed roles and procedures (Figure 1).

**Figure 2:**
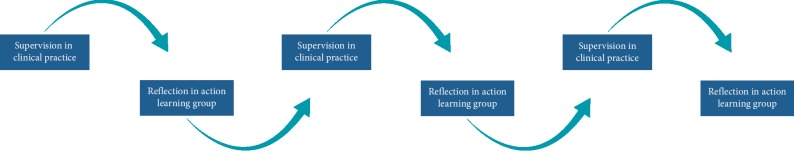
Back-and-forth movement between practice and reflection.

The framework in the action learning groups was structured as a cycle where each participant tries out the Model of Practical Skill Performance in clinical supervision (actions), reflects on the actions together with the participants in the action learning group (collective reflection), and commits to a new test of the model (new actions). Back-and-forth movement between action and reflection is central to this action learning concept (Figure 2).

At each meeting, one clinical supervisor offered to discuss her action. The facilitator asked investigative and appreciative questions about the performed action. The reflective team was invited to contribute with statements and reflections to illuminate the action and possibly produce new approaches and nuances to be used in future actions. The clinical supervisor had the opportunity to think out loud, move between closeness and distance to her experiences, and receive constructive feedback. Thus, the clinical supervisor and the action learning group could develop new ideas for future use and testing of the Model of Practical Skill Performance as well as choose targets for new actions.

During the action learning phases, the four project leaders were primarily engaged in securing data for the research project. Then, they offered supervision to the facilitators and groups if needed.

### 2.3. Data Collection

The project leaders collected data for the present study before, under, and after the action learning activities. Data triangulation was performed [25] by including data from questionnaires, notes, focus groups, and individual interviews with critical voices.

Participants received a questionnaire at baseline and after the project period including two questions: (1) To which degree do you understand the Model of Practical Skill Performance? and (2) To which degree do you use the Model of Practical Skill Performance in your own work as a clinical supervisor? Answer categories were very much, much, some, a little, and not at all. Furthermore, the questionnaires provided an opportunity for qualitative comments. The questionnaires were sent by e-mail to 129 participants before the meetings in action learning groups started, and 79 were returned (61.2%). The questionnaires were sent again to the 110 participants who still had their institutional e-mail ½-1 year after project termination. Forty questionnaires were returned (36.4%). Questionnaires were returned by e-mail or ordinary mail.

Notes were written by members of the group after each meeting. The aim of the notes was to gather their experiences from the meetings and to strengthen the groups' understanding of their own learning processes. In the notes, the groups described (1) the supervision situation they reflected on, (2) how they assessed the dialogue in the action learning group, and (3) how they assessed their level of understanding and use of the Model of Practical Skill Performance.

Three focus group interviews were held at the end of the project period. Focus group interviews were chosen to provide a deeper insight into promoting and inhibiting aspects of the action learning process as well as the participants' overall assessment of what they had gained from the project. Focus group interviews facilitate dialogue between participants that can stimulate memory and associations in relation to the implemented action learning project [27, 29, 30]. Participants were asked to volunteer for the interviews, and the three groups were formed with 6 and 8 clinical supervisors and 10 facilitators. An interview guide was developed with open questions targeting the elements in the action learning concept: experiences with the process, dialogue, interaction in the group, and confidence with and impact of the model on participants' supervision practices with students. All interviews were conducted by two of the project leaders and lasted between 45 and 60 minutes. As focus group discussions may be biased by dominant individuals and group thinking where members tend to maintain group cohesion [31], we had special focus on a few critical voices during the interviews.

In addition, three critical voices were contacted for follow-up in individual telephone interviews. In the interviews, these participants were invited to add to the opinions expressed in the focus group interview. All telephone interviews were conducted by one of the project leaders and lasted between 12 and 15 minutes. All interviews were audiotaped, and key passages were transcribed.

### 2.4. Data Analysis

The project leaders used deductive content analysis to analyze the qualitative data from questionnaires, notes, focus interviews, and telephone interviews [25, 32]. Deductive content analysis was chosen because we aimed to explore how clinical supervisors experiencing that action learning promoted their use of the Model of Practical Skill Performance under supervision of nursing students. The core concepts from action learning were used as lenses during the analysis: (1) reality close action, (2) alternation between action and reflection, (3) learning in community, and (4) personal ownership [26, 27]. Step 1: all four project leaders separately coded and categorized the data from questionnaires, notes, focus interviews, and telephone interviews related to these theoretical core concepts. Step 2: the whole group discussed and reached consensus on the data's categorization and validated findings by moving back and forth between the text and categories. Step 3: the texts were read again to identify promotional and inhibitory factors in the action learning process and their impact on the implementation of the Model of Practical Skill Performance as a supervision tool. Step 4: together, the four project leaders identified central citations from the data to illustrate connections between data and categories and increase the trustworthiness of the analysis. The in-depth dialogue between the four project leaders/researchers knowing the data in detail may promote content validity in the analyses.

The quantitative data in the questionnaires were analyzed with simple descriptive statistics (Table 2).

### 2.5. Ethical Considerations

According to the Danish law, permission to conduct the project was not needed from the Central Denmark Region Committee on Health Research Ethics. This study was approved by head nurses in participating departments. Participants were verbally informed about the project, and the data collection activity took place in parallel with the action learning process. Anonymity and confidentiality were ensured in the handling of data.

## 3. Findings

The object for implementation was the Model of Practical Skill Performance as a supervision tool for clinical supervisors. The aim of this study was to investigate the impact of action learning as an implementation method on the clinical supervisors' perception of value, impact, and sustainability of using this tool. Findings are therefore organized into two parts: First, we describe their perceptions according to value and impact of participating in action learning groups and how they viewed benefits and disadvantages in relation to the core elements in action learning: reality close action, alternation between action and reflection, learning community, and personal ownership. Second, the impact and sustainability of the participants' learning outcomes regarding understanding and use of the Model of Practical Skill Performance in clinical supervision are described.

### 3.1. Reality Close Action

It was expected that all clinical supervisors used the model between the meetings in the action learning groups as a supervision tool in their work in hospitals or primary health care. Opportunities to apply the model were many: introducing students to new skills as well as observing, assessing, and supervising students while they practiced the skills. In their daily practice of nursing, they realized that supervision of specific practical skills could be focused and qualified by using the model. One supervisor (clinical supervisor, primary health care) said, “I became aware that I had a new useful tool in my pedagogical toolbox.” Development of the supervisors' pedagogical skills was triggered by reflection on authentic actions.

In a few action learning groups, reflections on the actions stayed in the background in favor of a more theoretical discussion to enhance understanding of the Model of Practical Skill Performance or the action learning concepts (Figure 1). To gain familiarity with the Model of Practical Skill Performance, participants worked with various text materials and videos. Other groups spent a lot of time discussing questioning techniques and reflection methods. However, the approaches in these groups tended to inhibit the implementation of the model, as they were late in testing actions and only a few actions became the focus of collective reflections. One interviewee (clinical supervisor, medical hospital ward) said, “The clinical supervisors did not know the Model of Practical Skill Performance quite well, so we often discussed how to understand the model and asked the project leaders many questions. Clinical supervisors learned a lot from the process, but they did not use the model so much.” These groups kept a distance from specific clinical supervision situations, and it was up to each participant to transform the Model of Practical Skill Performance from a tool for theoretical reflection to a tool for clinical practice. These groups did not succeed in following the core concept of reflecting on reality close actions. It seemed that even though the nurses were competent clinical supervisors, some of them needed to become confident with the model before they could use it.

### 3.2. Alternating between Action and Reflection

In most action learning groups, all participants presented actions at the meetings. Participants experienced benefits from both reflecting on their own actions as well as listening to and reflecting on others' actions and reflections. Reflections in the group contributed to greater familiarity with the categories in the model and supported the supervisors' sense of security and courage to test it in new situations with their students. They picked up ideas on how to overcome barriers when using the model, and they described that they had gained familiarity with the model by applying it in their own actions. For example, one supervisor (clinical supervisor, medical hospital ward) said, “Interaction between the reflection in the group and testing own actions, made me more confident, even when I had to discuss with students who were critical of the model.”

The clinical supervisors' personal goals for their own actions were important, and group discussions contributed towards setting realistic individual goals for new actions. One supervisor (clinical supervisor, primary health care) said, “I was jointly evaluated at the last meeting, and I realized how confident I was using the model in my supervision. After the meeting I wrote a letter to myself about my own new goal, about using it together with the students in the coming clinical placement period.”

### 3.3. Learning Community

The majority of the groups reported that they had an open process in the groups. They described the need for transparency and trust as central elements in the process of discussing something that was new and challenging. Prior knowledge of one another could be conducive to the process, but there were also examples of the opposite. Group members described that sharing experiences from different contexts promoted a genuine interest in one another's experiences (clinical supervisors, medical hospital ward), “We did not know one another, but it was inspiring to come from different places. We asked sincere questions to the actions of others and we quickly developed a dynamic dialogue, and everyone showed interest in the other's actions.”

The obligation to participate in the learning community was shown through active engagement in the different roles that were expected of them according to the structured setup in the action learning groups. Participants collaborated in planning actions that could be the subject for discussion and reflection in upcoming meetings. However, some groups were challenged in their collaboration to follow the action learning process, for example, when former power relations from previous cooperation were transferred to the action learning groups. Discussion about everyday problems in the workplace could also steal time from reflection and discussion. A strategy was to allow time for small talk in the beginning before moving on to the study agenda. This seemed to reduce the challenge of focusing on their own specific cases of supervising with the Model of Practical Skill Performance.

Many participants experienced that the tight structure of the action learning concept increased their participation in the reflection processes. It was important that all participants presented their actions because reluctance to speak about their own experiences seemed to reduce the member's commitment. When the action learning concept was not followed, the meetings were typically organized as roundtable discussions, which weakened the group's focus on the Model of Practical Skill Performance and reduced the members' personal ownership and sense of commitment towards the implementation of the model.

Some participants experienced that the project period was too short to become familiar with the model and to test it in practice. Other participants felt that there was too much time between meetings, which made it hard to stay focused on the model as many other work-related issues demanded their attention. A few groups contacted the project leaders because they needed to discuss the action learning concept, interview techniques during meetings, or management of the reflection process.

### 3.4. Personal Ownership

To benefit from participation in learning groups, personal ownership of the Model of Practical Skill Performance seemed important. Several nurses who had voluntarily chosen to spend time and energy on the project expressed that participation gave them a coveted opportunity to spend time to focus on quality and gain a deeper understanding of clinical education in nursing. One nurse (clinical supervisor, surgical hospital ward) expressed, “It's fantastic to get the opportunity to be deeply involved in a specific pedagogical supervision method… usually we have not got the time for reflection or systematic testing of learning tools.” The motivation and commitment among participants were stimulated by the kick-off seminar, especially the presentation of the model by the researcher who had developed it. One nurse in a focus group (clinical supervisor, medical hospital ward) said, “At first I was skeptical but... all my resistance vanished, that was a good start.”

Some supervisors did not participate voluntarily but were strongly encouraged to participate by their head nurse. Their reservations were expressed in their behavior in the clinical setting and in the groups. They did not use the model and had no cases to present, and they hardly participated in reflections in the learning group. A few groups were dominated by nurses with weak ownership and commitment, and this resulted in unprepared participants and few substantial discussions. The benefits of action learning in these groups thus seemed sparse.

### 3.5. Learning Outcomes: Understanding the Model of Practical Skill Performance

Prior to participating in this project, the understanding of the model differed between participants (Table 2). Despite participation in earlier introduction to and reading about the model, the clinical supervisors described uncertainty in relation to understanding the categories in the model. The reflection on supervision in practice in the action learning groups had helped them to distinguish between the categories in the model and to acknowledge the complexity of practical skill performance in general. Learning outcomes of the project seemed to last over time; six to 12 months after project completion, 70% of the clinical supervisors who answered the questionnaire indicated that they understood the model “much” or “very much” (Table 2).

### 3.6. Learning Outcomes: Using the Model in Clinical Supervision

Before participation in the project, more than 60% of the supervisors who answered the questionnaire reported that they had never or only a few times used the model during clinical supervision (Table 2). Some had tried to use the model but believed they were unsuccessful and that it was time-consuming and cumbersome. The main barrier for applying the model seemed to be their own uncertainty (clinical supervisor, medical hospital ward) about the model and its categories, “I have used it in supervision, and however, I feel uncertain about some categories; for example the category integration. As a matter of fact, I do feel a little sketchy in my use of the model.”

Participation in the project created familiarity with the Model of Practical Skill Performance. The format of the meetings in the action learning group helped many to become more familiar with the model by “pushing” them to “jump into it” as well as to plan and present an action while using the model for discussion and reflection. The discussions gave them a common language about practical skill learning they could use with students and colleagues. Many clinical supervisors expressed that the quality of their supervision improved during the project. One supervisor (clinical supervisor, surgical hospital ward) declared, “I use the model in my pre- and post-supervision. I use it to promote the students' reflection. I think my instructions have become more qualified and targeted.”

The supervisors' enhanced qualifications benefitted the students' learning through more structured supervisions and detailed reflections. They used the Model of Practical Skill Performance during the introduction of new students to their clinical placement and offered the students the opportunity to use the model to observe the nurses' own practice. Students were positive and readily accepted the model as a learning tool when initial critique had been addressed. Table 2 shows that, after the completion of the project, only 12.5% of the participants who had completed the questionnaire still did not use the model during supervision.

A few clinical supervisors reported that they had little benefit from participating in the action learning groups and felt that too much time was spent on the project compared to the outcome. Some described the ordinary bustle of work as a barrier to implementing the model in their ward. Others stated it was hard to give the model special attention because use of the model was in competition with other time-consuming activities during clinical supervision.

## 4. Discussion

Focus of the discussion will be on the potential of this action learning setup as an implementation method in clinical practice, aiming at changing the practice of clinical supervisors. Our findings about the process and outcome will be discussed retrospectively, in light of the Promoting Action on Research Implementation in Health Services (PARIHS) framework [33, 34]. The PARIHS framework is chosen in this phase of the project to discuss the findings although it is frequently used to guide development and evaluation of the aspects of implementation processes. The PARISH framework focuses on evidence, context, and facilitation as essential core elements in an implementation process [33]. The framework can be used both retrospectively and prospectively to understand how these core elements impact on implementation of new interventions [34].

### 4.1. Evidence

The PARISH framework highlights that quality of the evidence for implementation is considered important for the success of an implementation process [33–35]. Evidence needs to be robust, and users have to believe in the evidence [36]. The evidence implemented in this study was the Model of Practical Skill Performance, a theoretically and empirically based model [14]. Although baseline data showed that many participants did not understand the model very well, focus group interviews revealed that most participants considered the model to be intuitively relevant to their supervision practice. No one was openly critical to the model during the introduction or during the project as a whole. The introduction to the model by its creator and knowledge about its relevance obtained from former research [18–21] might have increased participants' acceptance of the model for use in clinical supervision and thus supported the implementation [34]. The clinical supervisors were encouraged during their implementation of the model when students were receptive and eager to use the tool. This supported their experience of the model as “good” evidence. According to Rycroft-Malone et al. [36], robust evidence is not enough; a crucial promoter is positive response from individuals involved in the implementation process.

The structure and agreement about the rules for action learning required a certain behavior from the participants in terms of action and reflection [27]. The movement between action and reflection (Figure 2) could potentially bridge the gap between the theoretical model and its practical use, thereby strengthening the relevance of the evidence or the model. Low activity with the model during supervision practice and prolonged discussions about the model's complexity in a few groups might indicate distrust towards the Model of Practical Skill Performance and its usability. Research knowledge is seldom possible to apply without some sort of vetting or tailoring to the local context. Although the model has been accepted and used successfully in other contexts, it is important to assess local barriers to knowledge such as not understanding the knowledge to be implemented [35].

### 4.2. Context

The PARISH framework focuses on how contextual readiness can promote or inhibit the change [33]. In the present project, both action learning groups and local clinical practices represent the context, although the major part of our findings on the context was related to the action learning groups. Stetler et al. [33] defined aspects of context as culture, evaluation capabilities, leadership support, and receptivity to the innovation. The culture in the action learning groups was created by the participants' values and beliefs expressed during the meetings. Most of the clinical supervisors demonstrated a positive attitude to the implementation process and had the courage and communicative skills to engage in different roles such as presenting the action of testing the model, being the interviewer, or being the member of the reflective team. Implementation is promoted when practitioners are given specific tools and education that can underpin new behavior [34, 37]. In this project, the structured reflection process (Figure 1) was viewed as a specific tool. It was thus a promoting factor that all participants had a short pedagogical education and were familiar with reflection as an important part of learning. Overall, the participants expressed it as valuable and inspiring when the group focused on reflection on concrete actions. Similar findings are described by Machin and Pearson [22], who studied action learning to promote practice and leadership development among new nurses and midwives.

In the few groups where meetings were focused on discussions of the Model of Practical Skill Performance, it seemed these discussions challenged the action learning process by shifting the attention away from the actual experience of using the model. Theoretical input during action learning can be viewed as valuable [5], but in this project, the focus on theory reduced participants' opportunity to create knowledge from real-life actions where they applied the model during clinical supervision. A few clinical supervisors were not receptive to the ideas of the project and seemed to sustain their skepticism towards the model or the implementation method throughout the whole project period. It may be a barrier that a few participants did not have the courage or were unwilling to be introspective and to expose themselves and their mode of clinical supervision to the action learning group. Another barrier could be related to the context of their workplace with a high workload, which might decrease creativity and ingenuity in supervision. A barrier in the implementation could be a lack of clarity related to the authority to change practice. This could be the case when some clinical supervisors talked about being the only one focusing on the model in their department. Another barrier in the action learning concept could be related to some supervisors' wish for concrete instructions instead of being active in deciding and creating learning situations [37].

By allocating resources to the clinical supervisors' participation in action learning groups, the project was generally supported by leaders in the clinical setting. However, the findings also showed that participants found it difficult to allocate time in their daily work to meet the obligations related to their involvement in action learning groups. These may be typical barriers for changing ongoing practice but underline the importance that leaders must be aware of their power to promote implementation of projects in clinical practice [37].

### 4.3. Facilitation

As highlighted in the PARISH framework, the project confirmed the importance of the role and competences of the facilitators in the implementation process [33]. The facilitators can be characterized as “innovators” or “early adopters” [38]. The facilitators had a positive approach to innovation in nursing education in general, including use of the Model of Practical Skill Performance, and they participated in the seminar to prepare their role in the action learning groups. In spite of the facilitators' competence and preparation, the structure for group reflection was not maintained in all settings. It could have helped the process in those groups which spent a considerable amount of time on theoretical discussions if the facilitators were more strict in adhering to the structured reflection process on chosen actions [27, 28].

Some facilitators allowed for small talk about everyday problems from the participants' workplace in the beginning of each meeting, followed by the described structure for group reflection. This approach seemed to support the social processes in the groups and helped the group to settle and to concentrate on the action learning processes and the discussion of how they had used the Model of Practical Skill Performance in action. This action learning setup was based on the individuals' behavior while learning in the reflection group; nevertheless, power relations or passivity from individuals impacted negatively on the team climate. Rycroft-Malone et al. [36] highlighted the impact of individual motivation and self-efficacy about changing practice; in this study, these aspects might have been underestimated. It could be difficult for the facilitators to confront passivity and find solutions on how to overcome possible vulnerability causing passivity among some clinical supervisors. Relationship building can be very important to promote a safe atmosphere where it is acceptable to expose one's actions and thoughts [22]. Participating and exposing oneself in action learning groups can be challenging and overwhelming for the individual, but in the end, the result is deeper personal growth [39].

### 4.4. Limitations

Fewer questionnaires were mailed to the participants after the action learning period due to the change of job among supervisors; thus, fewer participants had the opportunity to complete the second questionnaire. The supervisors' high degree of understanding and use of the Model of Practical Skill Performance in supervision practice must therefore be interpreted with some caution. Action learning projects are ideally driven by participants who are engaged in the content of a project, and most participants in this study were positively engaged and interested in the use of the model. This might mean that although we located and interviewed three participants with critical voices, a different recruiting process might have included participants with more varied views on the use of the model.

## 5. Conclusion

This project explored action learning as a method to implement the Model of Practical Skill Performance as a learning and supervision tool in clinical education of nursing students. The findings suggest that the implementation method was mostly experienced as positive. An important reason why the action learning method was successful can be the dedication of facilitators who were competent in independently leading the action learning through a structured reflection process based on the group members' experiences with actions; this seems to have promoted the use of the Model of Practical Skill Performance in the clinical setting. Action learning was mainly experienced as an engaging and effective method to implement the supervision tool. The main strength seemed to be the autonomous local group that supported collective reflections on actions.

The findings of this study support the use of a large-scale action learning project to promote the dissemination and use of a tool for nursing student supervision in clinical practice. The results imply that collaboration over time between stakeholders in education, practice, and management is necessary to implement changes that have long-time consequences. Action learning is based on the members' willingness to both act and reflect on the topic of learning; this implies a need for clinical managers to secure voluntariness and motivation when choosing participants to join in such projects. The positive change in participants' scores on understanding and use of the model implies that involving clinical nurses in long-time participation may be a very important factor when planning projects that aim at the permanent change in practice. The project was driven by faculty and clinical educators with both research experience and intimate knowledge on the topic of implementation. The positive results indicate that such knowledge is imperative when launching projects that involve both education and practice.

## Figures and Tables

**Table 1:** Instrumental supplement to the Model of Practical Skill Performance (http://www.rins.dk).

Definition of categories in the model	Characteristics of quality performance
*Substance* and *sequence* are the core aspects of a practical skill. They imply that necessary steps in the skill are included and performed in a logical order.	Substance and sequence are determined on the basis of the content in clinical guidelines, professional standards, and principles. Substance and sequence are adapted to the patient and the situation where the skill is being performed.

*Accuracy* refers to exactness of each movement step, instruction, and information. Accuracy is important in order to ensure security of the patient, nurse, and environment.	Accuracy implies to act (i) Correctly (ii) Precisely Accuracy implies to inform and instruct (i) What is necessary and sufficient (ii) Distinctly (iii) Understandably

*Fluency* signifies that tempo and rhythm are adjusted to both the patient and the type of the practical skill being performed and that the practical skill is performed with smoothness.	Fluency implies to act, inform, and instruct (i) Without hesitancy (ii) Without unnecessary breaks (iii) With ease

*Integration* signifies that all parallel aspects within the practical skill are harmonized. Integration also means that the practical skill, as a whole, is adjusted to the patient's current condition and situation.	Integration implies to (i) Time and coordinate the elements of action Integration related to adjustment implies to (i) Be attentive (ii) Have an overview (iii) Be flexible

*Caring comportment* signifies to create an atmosphere where the patient's dignity is upheld, self-determination is ensured according to the patient's current condition and situation, and well-being is warranted.	Caring comportment implies to (i) Acknowledge (ii) Show respect (iii) Ensure patient participation (iv) Be empathic (v) Use appropriate touch (vi) Be engaged (vii) Use appropriate communication (viii) Work aesthetically

**Table 2:** Understanding and use of the Model of Practical Skill Performance.

	Very much, %	Much, %	Some, %	A little, %	Not at all, %
Understanding the model					
Before, *n* = 79	1.3	11.4	36.7	39.2	11.4
After, *n* = 40	7.5	62.5	27.5	2.5	0

Extent of use of the model					
Before, *n* = 79	1.3	5.1	29.3	35.2	29.1
After, *n* = 40	5	27.5	55	0	12.5

## Data Availability

The data from notes, audiotaped interviews, and questionnaires used to support the findings of this study are available from the corresponding author upon request.
